# l-Aspartate: An Essential Metabolite for Plant Growth and Stress Acclimation

**DOI:** 10.3390/molecules26071887

**Published:** 2021-03-26

**Authors:** Mei Han, Can Zhang, Peter Suglo, Shuyue Sun, Mingyao Wang, Tao Su

**Affiliations:** 1 Co-Innovation Center for Sustainable Forestry in Southern China, College of Biology and the Environment, Nanjing Forestry University, Nanjing 210037, China; sthanmei@njfu.edu.cn (M.H.); can18723@126.com (C.Z.); petersuglo@yahoo.com (P.S.); sunsyjessica@163.com (S.S.); njfuwmy2021@163.com (M.W.); 2 Key Laboratory of State Forestry Administration on Subtropical Forest Biodiversity Conservation, Nanjing Forestry University, Nanjing 210037, China

**Keywords:** aspartate, stress, aspartate aminotransferase, aspartate transporter/carrier, compartmentation, hormone

## Abstract

L-aspartate (Asp) serves as a central building block, in addition to being a constituent of proteins, for many metabolic processes in most organisms, such as biosynthesis of other amino acids, nucleotides, nicotinamide adenine dinucleotide (NAD), the tricarboxylic acid (TCA) cycle and glycolysis pathway intermediates, and hormones, which are vital for growth and defense. In animals and humans, lines of data have proved that Asp is indispensable for cell proliferation. However, in plants, despite the extensive study of the Asp family amino acid pathway, little attention has been paid to the function of Asp through the other numerous pathways. This review aims to elucidate the most important aspects of Asp in plants, from biosynthesis to catabolism and the role of Asp and its metabolic derivatives in response to changing environmental conditions. It considers the distribution of Asp in various cell compartments and the change of Asp level, and its significance in the whole plant under various stresses. Moreover, it provides evidence of the interconnection between Asp and phytohormones, which have prominent functions in plant growth, development, and defense. The updated information will help improve our understanding of the physiological role of Asp and Asp-borne metabolic fluxes, supporting the modular operation of these networks.

## 1. Introduction

l-aspartate (Asp), in addition to constituting proteins and being an active residue in many enzymes, is a precursor leading to the biosynthesis of multiple biomolecules required for plant growth and defense, such as nucleotides, nicotinamide adenine dinucleotide (NAD), organic acids, amino acids, and their derived metabolites. Though it cannot be simply quantified, given that in *Escherichia coli*, approximately 27% of nitrogen flows through Asp (https://MetaCyc.org, accessed on 30 January 2021) [1], the contribution of Asp to plants is highly conspicuous. It has been well documented that methionine (Met), threonine (Thr), lysine (Lys), and isoleucine (Ile), of the eight essential amino acids, are derived from Asp, through a pathway commonly known as the Asp family amino acids [2]. Further metamorphosis of Asp can yield glutamate (Glu) to glutamine (Gln) through the action of glutamine synthetase (GS). Asp and Glu, along with asparagine (Asn) and Gln, are the common nitrogen carriers [3], which have been noted for their primary role in the recycling, storage, and transport of nitrogen in germinating seeds, vegetative organs, and senescence organs [4]. Asp is also involved in the biosynthesis of some other amino acids such as arginine (Arg) and the aromatic amino acids (tyrosine (Tyr) and phenylalanine (Phe)), through the aspartate–argininosuccinate synthase and the aspartate–prephenate aminotransferase pathways, respectively [5]. Moreover, Asp is the building block for de novo pyrimidine manufacturing and is required to convert ionosine-5′-monophosphate to adenine-5′-monophosphate in purine biosynthesis [6]. In addition, Asp serves as a critical precursor of the aspartate oxidase pathway in the synthesis of nicotinamide adenine dinucleotide (NAD), an essential component of plant abiotic process, senescence, chlorophyll formation, and pollen development [7,8,9]. In addition, Asp deamination to oxaloacetate by aspartate aminotransferase (AspAT) in the cytosol is essential for the production of malate needed in mitochondria for the tricarboxylic acid (TCA) cycle [10], whereas Asp released from the mitochondrion is involved in the biosynthesis of nucleotides in the cytosol. Intriguingly, some recent studies have found that cytosolic Asp is an endogenous metabolic limiter of cell proliferation [6,11,12,13,14,15], moreover, Asp derived from glucose is indispensable to drive biomass synthesis during cellular hypertrophy [16]. Altogether, apparently, Asp represents a critical metabolite hub interconnecting with diverse metabolic pathways that are of significant importance for plant nutrition, energy, and stress responses.

**Figure 1:**
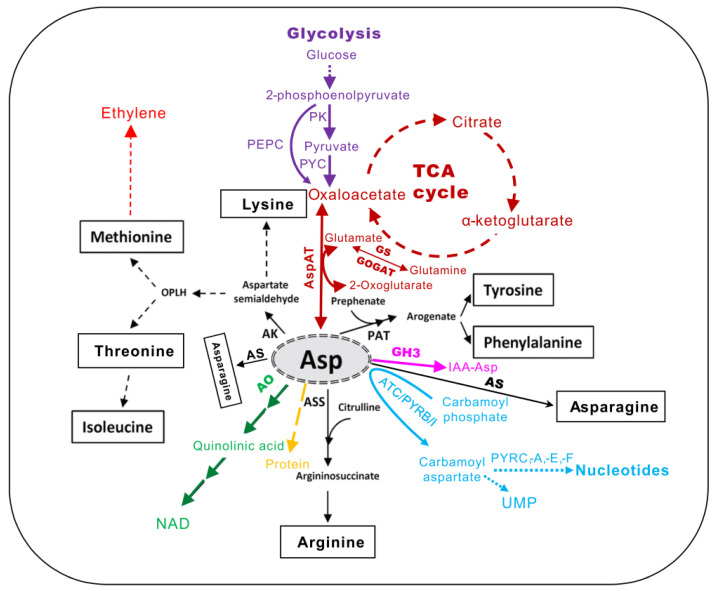
The central metabolic intermediates derived from l-aspartate (Asp) in plants (adapted from [5]). AK, aspartate kinase; AO, aspartate oxidase; ASS, argininosuccinate synthase; AS, asparagine synthase; PAT, prephenate aminotransferase; AspAT, aspartate aminotransferase; GS, glutamine synthetase; GOGAT, glutamine oxoglutarate aminotransferase; TCA, tricarboxylic acid cycle; NAD, nicotinamide adenine dinucleotide; PK, pyruvate kinase; PYC, pyruvate carboxylase; PEPC, phosphoenolpyruvate carbosylase; ATC/PYRB/I, aspartate transcarbamoylase or aspartate carbamoyl transferase; PYRC, dihydro-orotase; PYDA, dihydro-orotate dehydrogenase; PYRE, phosphoribosyl transferase; PYRF, orotate decarboxylase. GH3, group II of GRETCHEN HAGEN3 family of acyl amido synthetases.

Exchange and competition for Asp and derived intermediates profoundly affect plant metabolism, which requires great attention. The detailed study and research into anabolism and catabolism of Asp and its related pathways (i.e., the Asp family amino acids, nucleotides, NAD, TCA, and glycolysis) are thus necessary to increase our knowledge on cell growth and repair [17], so as to further our understanding of plant growth, development and defense [13,15,18]. Herein, the various pathways derived from Asp are summarized in this review (Figure 1), and a general overview of Asp metabolism and regulation is described. In addition, the dynamism of Asp and AspAT in plants and their role in the plant in response to various stress conditions are discussed. Furthermore, some recent progress in the interconnection between Asp and phytohormones, such as ethylene and auxin, is highlighted.

## 2. The Biosynthesis and Transformation of Asp in Plants

The biosynthesis of Asp is the interface between amino acid metabolism and carbohydrate metabolism (https://MetaCyc.org, accessed on 30 January 2021) [1]. The carbon for de novo Asp synthesis is provided by anaplerotic Glu, which converts to α-ketoglutarate (also known as 2-oxoglutarate (2-OG)) by either glutamate dehydrogenase (GDH) or transamination along with electron pair transfer [11,19]. Alternatively, an increase in glucose consumption but not Gln is found to be essential for the anabolic pathway of Asp, and glucose-derived Aspenhanced de novo nucleotide synthesis, which is crucial to driving biomass synthesis during cellular hypertrophy [16].

### 2.1. Key Enzymes Involved in Asp Anabolism and Catabolism

#### 2.1.1. Aspartate Aminotransferase

Regardless of the differences in the origin of the carbon source, Asp is synthesized mainly from and degraded to oxaloacetate through a transamination reaction mediated by the enzyme aspartate aminotransferase (AspAT, E.C. 2.6.1.1., also known as glutamate–oxaloacetate transaminase (GOT)), an important aminotransferase present in all of the free-living organisms [20,21]. AspAT is a much-conserved enzyme found in both prokaryotes and eukaryotes. It channels nitrogen between Glu and Asp [4,5] and is closely linked to purine’s biosynthesis salvage pathway as well as the glycolytic and oxidative phosphorylation pathways [22].

In plants, the AspAT enzymes exist in multiple forms and are mostly localized into three subcellular compartments, associated with the cytosol, mitochondria, and plastids [5,23,24,25,26]. *Arabidopsis* contains a family of five genes encoding distinct AspAT isoenzymes. *AtAspAT1* encodes mitochondrial isoenzymes and *AtAspAT2* and *AtAspAT4* encode cytosolic isoenzymes, whereas *AtAspAT3* encodes a putative peroxisomal or plastid isoenzyme and *AtAspAT5* encodes the plastid isoenzymes [27,28]. All these *AspATs*, independent of their subcellular location, originate from the Iα subfamily. However, a novel form of plastid AspAT found in all plants was reported to exhibit subfamily Iβ enzyme properties from prokaryotes [29], meaning that the plastids contain both the eukaryotic (e.g., *Arabidopsis* AspAT5) and the prokaryotic form of AspAT. The latter is a bifunctional enzyme possessing both AspAT activity and prephenate aminotransferase (PAT) activity (hereafter named AspAT/PAT).

#### 2.1.2. Asparate Kinase (AK)

Aspartate kinase (AK) is the first and the most crucial enzyme in the Asp-derived amino acid pathway in the plastid. It catalyzes the phosphorylation of Asp, the first reaction that leads to the biosynthesis of four essential amino acids: Met, Thr, Lys, and Ile [30]. The concentration of Lys and Thr mediates the catabolism of the Asp, which indirectly affects the synthesis of Met and Ile in plants [31,32]. It is difficult for scientists to breed crops accumulating a large amount of one of the amino acids mentioned above since they in turn control the breakdown of Asp. To effectively produce crops with a large amount of any of these Asp family amino acids, the Asp metabolic flux arrangement (holistic approach) has to be considered.

#### 2.1.3. Aspartate Oxidase (AO)

Aspartate oxidase (AO; EC 1.4.3.16) catalyzes the first irreversible reaction in NAD synthesis in many bacteria and plants. It is a B protein of quinolinate synthetase (QS), which was first discovered in *E. coli* [33]. In plants, de novo synthesis of NAD starts with the oxidation of Asp to α-iminosuccinate by the action of AO, and thereafter QS, and quinolinate phosphoribosyltransferase (QPT) catalyzes the next reactions to yield quinolinic acid and nicotinate mononucleotide, which finally undergo further enzymatic changes to produce NAD in the plastid [34,35,36]. NAD is a key molecule in intermediary metabolism in many plants’ physiological processes, including senescence, chlorophyll synthesis, pollen development, sensitivity to abscisic acid (ABA) levels, drought, ultraviolet radiation b (UVB), salinity, and heat shock [7,8,9]. NAD also acts as a co-factor in energy transformation and electron transfer. Thus, manipulation of Asp in the plastid would likely significantly impact NAD synthesis and the general metabolism of substance and energy in plants.

#### 2.1.4. Argininosuccinate Synthase (ASS)

Asp catabolism in the plastid plays a vital role in the synthesis of Arg, which can further be catabolized to urea and finally degraded to form carbon dioxide and ammonia by the cytosolic urease. It has been reported that the fourth N-atom of Arg is generated from Asp, which is ligated to citrulline by argininosuccinate synthase (ASS). Argininosuccinate lyase (AL) splits off fumarate, yielding the final product Arg [37]. In short, ASS catalyzes the synthesis of arginosuccinate from Asp and citrulline, which is subsequently converted into Arg through AL [38]. The catabolism of Asp, leading to the anabolism of Arg, is essential for plants’ defense against abiotic and biotic stress. It provides precursors for many signaling molecules such as nitric oxide (NO) and polyamines during stress [39].

#### 2.1.5. Aspartate Transcarbamylase (ATC)

Aspartate transcarbamylase (ATC or ATCase), also known as aspartate carbamoyltransferase, is a ubiquitous enzyme that catalyzes the initial reaction between Asp and carbamoyl Asp to form N-carbamoyl-aspartate in the pyrimidine biosynthesis pathway [40]. The larger part of the pyrimidine molecule (C4-C5-C6-N1) comes from the Asp, which shows the critical role of Asp in DNA and RNA biosynthesis, and finally in cell multiplication and repairs [41]. In plants, the first two enzymatic reactions of the Asp leading to pyrimidine biosynthesis are localized in the plastid, but the rest of the reaction is located outside the plastid, with all the steps being encoded by single genes [42,43].

#### 2.1.6. Malate–Aspartate Shuttle

A critical role of Asp is that it transfers reduction equivalents produced during glycolysis across the mitochondrion membrane for oxidative phosphorylation in eukaryotes to generate ATP through the malate–aspartate shuttle pathway. The malate–aspartate shuttle consists of AspAT, and malate dehydrogenase (MDH), which converts malate to Asp in the mitochondrion, and the reverse reaction occurs in the cytosol [44]. Asp is transported to the cytosol via specific Asp carriers, increasing the NAD+/NADH ratio when converted to malate by AspAT2 and MDH2 [45]. The Asp’s availability in cytosol depends on the Glu/Asp carriers mediated by ions like Ca^2+^ [44]. At a lower Ca^2+^ level, the malate–aspartate shuttle activities are stimulated, while at a higher level, α-ketoglutarate dehydrogenase is activated in the mitochondrion matrix, resulting in a limitation of the α-ketoglutarate for the malate–aspartate shuttle [46]. In the cytosol, oxaloacetate is synthesized by the AspAT, which is finally reduced by NADH to malate. Malate is then transported across the mitochondrial membrane by the malate-α-ketoglutarate carrier to be oxidized by NAD+ back to oxaloacetate [47]. The NAD+/NADH ratio is lower in the mitochondrial matrix than the cytosol, causing an imbalance in the pathway and therefore repeating the process [44].

### 2.2. Aspartate Transporters

Asp synthesis and degradation pathways are compartmented [24,48]; thus, transport between various intracellular compartments (i.e., chloroplast, mitochondrion, and cytosol) is essential for Asp activity. Transporters play an essential role in distributing molecules to regulate physiological processes connected to a specific cellular demand and causing balance for effective growth and development. Asp transporters, just as any transporter, play a significant role in the distribution and the balance of Asp in plants and other organisms. For instance, Asp’s transport from the mitochondrion to the cytosol is necessary for nucleotide formation and other metabolites for cell proliferation [13].

Aspartate-glutamate carriers (AGCs) are types of mitochondrial carrier family which are responsible for the mitochondrial exportation of Asp [49]. The malfunction of AGC results in a significant drop in the proliferation of several cell lines [15,50]. In contrast, mitochondrial respiration is stimulated by AGC1 activation [49]. However, to date, no ortholog human AGCs have been reported in plants [51]. Human uncoupling proteins (UCP2, UCP5, and UCP6) are other types of Asp transporter found in the mitochondrial membrane [52], among which UCP2 shows high protein identity with *Arabidopsis* AtUCP1 and AtUCP2 (51% and 45%). The primary function of UCPs is to allow the exchange of Asp in and Glu out through the mitochondrial membrane [51]. UCPs also play a crucial role in nitrogen metabolism due to their ability to exchange amino acids in various dicarboxylates [53,54].

The *Arabidopsis* AtUCP1 and AtUCP2 (also called PUMP/AT3G54110 and PUMP2/AT5G58970, respectively) are notably the first reported mitochondrial carriers transporting Asp and Glu in plants. These transporters also associate with malate–aspartate shuttle (MAS) enzymes to export reducing equivalents of NADH from the mitochondrion [51]. AtUCPs equally transport Asp/Glu and cysteinsulfinate (Palmieri et al., 2001). AtUCP1 and AtUCP2 have sequence similarity of about 72%; however, they differ significantly in their specific activity, making AtUCP1 more active in the transport of Asp/Glu than AtUCP2 [51]. Additionally, a recent report shows that the knockout of *lysine–histidine-type transporter 1* (*OsLHT1*) in rice affected root uptake of Asp, which eventually impaired the nitrogen translocation to the shoot, suggesting that OsLHT1 could possibly be an Asp transporter as well [55]. Despite several putative Asp membrane transporters identified to date in different genome databases, their transport capabilities in plants are yet to be investigated. It remains to be seen whether and what other transporters in plants can take up Asp under natural or stress conditions.

### 2.3. The Effect of Asp/Asn Homeostasis on Plants

Asp plays an essential role in the biosynthesis of Asn, noted for its primary role in the recycling, storage, and transport of nitrogen in germinating seeds, vegetative organs, and senescence organs [4]. The relationship between Asp and Asn is closely linked to the catabolism of many metabolites. The efficient breakdown of Asp into Asn is essential for desired nitrogen supply, especially during seed germination. Meanwhile, the elevated ration of Asn to Asp is very critical for ammonium assimilation, stress, and syntheses of nucleotides and amino acids, as well as the supply of cellular energy [56,57,58]. An increase in the accumulation of Asp and a declined level of Asn have been observed when asparagine synthase (AS) (which catalyzes ATP-dependent ammonia to Asp, yielding Asn) was targeted by siRNA, which finally led to a decrease in the Asn to Asp ratio and thus affected cell survival [59]. In soybeans, the increase in the *AS1* levels in the leaves (source) is positively correlated with seed protein concentration (sink), and expression of *AS* genes is associated with the ratio of Asn to Asp in the leaves [60,61]. In *Arabidopsis*, the overexpression of *AS1* (thus more Asp catabolism) correlates with an increase in seed storage protein concentration due to the elevation of Asn levels, namely, the nitrogen levels [62,63]. Nevertheless, aspartate kinase (AK) (also called aspartokinase) is another enzyme that competes with AS for Asp, which, therefore, potentially reduces the availability of Asp for the synthesis of Asn, leading to the synthesis of Asp family amino acids [64]. On the other hand, Asp can be synthesized from Asn by the enzyme asparaginase (ASNase) [65], which is then used for amino acid biosynthesis [4]. In addition, an increase in Asp corresponds to a decrease in Asn in the knockdown mutant expression of the *Arabidopsis dihydrodipicolinate synthase* (*DHPS*) gene that leads to an increase in free Lys levels [66]. Hence, the appropriate ratio of Asp and Asn modulated by the effective balance between AK and AS at any stage of plant growth and in a specific tissue is essential for plant growth and defense and for the optimum accumulation of essential amino acids for human/animal nutrition.

## 3. Role of Asp in Growth and Stresses

### 3.1. Asp is an Endogenous Metabolic Limitation for Cell Proliferation

Cytosolic Asp has profound importance to the proliferating cells, as it determines the cell’s survival, especially when Gln is limited [13]. The drop in cytosolic Asp resulting from the knockdown of *aspartate–glutamate carrier 1* (*AGC1*, known as *ARALAR*) leads to the reduction of the proliferation of several cell lines [15]. On the contrary, the supply of exogenous Asp or overexpression of an Asp transporter can bypass the need for an electron transport chain to support cell proliferation [6], demonstrating that Asp biosynthesis is a golden requirement for cell proliferation [13]. This has been further confirmed by the finding that TCA can only fully restore cell growth if it partners with Asp biosynthesis, thus, when AspAT is activated [15]. Further DNA content analysis by propidium iodide staining and flow cytometry reveals that the requirement of Asp for cell growth is at least partially because it sustains nucleotide biosynthesis [11,13].

### 3.2. Asp in Plants Coordinates Nitrogen Assimilation into Amino Acids

Asp and Glu and their amides make up more than one-third of the free amino acids in *Arabidopsis* [3]. They link the in vivo metabolism of amino acids to the relevant organic acids in the TCA cycle and the carbon metabolism in the glycolysis pathway [67,68]. When carbon skeletons are limited, Asp is amidated to form Asn, which serves as an efficient nitrogen transport and storage compound due to its relatively high N:C ratio (2:4) [69,70]. Under nitrogen stress, Asp appears to be one of the most importantamino acids [71,72,73,74,75]. It has been found that when N is sufficient, as a predominant amino acid translocated in plant phloem, Asp supplied by the phloem is converted in the root to Asn to export N to the shoot via xylem as part of the process of nitrogen assimilation, whereas, when N is absent, Asp supplied by the phloem is diverted to the formation of malate to support the metabolism cycle back to the shoot [74]. In a very recent study, higher Asp and Asn contents were observed to be positively coordinated with the nitrogen use efficiency (NUE) trait in potatoes with low N supply [75]. The above results suggest that Asp is imperative for amino acid and organic acid biosynthesis, especially under fluctuating N conditions. Asp coordinates nitrogen assimilation into amino acids such that the available carbon skeleton is mobilized [27,76]. Further targeted regulation of Asp metabolism might be a useful strategy to improve the NUE traits in plants.

### 3.3. Asp is a Drought Stress-Specific Responsive Metabolite

One of the most critical processes that affects plants under drought conditions is the accumulation of solutes, including amino acids in the leaf tissues and the roots. Asp concentration was recorded to increase by more than twofold in drought treatment in *Brassica napus* [77], *Astragalus membranaceus* [78], and Triticeae [79]. Similarly, Asp has shown the second-highest concentration (the second most activated compound) after ABA in root exudates of the holm oak (*Quercus ilex*) upon drought treatment [80]. Additionally, in chickpea plants treated with a plant growth-promoting rhizobacterium (PGPR) and plant growth regulator (PGRs) consortium and grown under drought stress conditions, a higher accumulation of Asp in the leaf of the tolerant variety was recorded as compared to the sensitive variety [81]. In addition, a significant change of Asp has been recorded in kale [82] and *Caragana korshinskii* [83], though its content declined upon drought stress. Regardless, the great range of variation of Asp content upon drought exposure suggests that Asp can serve as a drought-responsive biomarker.

### 3.4. The Variation of Asp Level Is Closely Linked to Stress Acclimation

When exposed to stress, plants accumulate a multitude of metabolites, particularly amino acids. A line of studies suggest a close correlation between the variation of Asp content and plant stress [84]. For example, under alkaline salt stress, a significant increase (3.97-fold) in Asp and other metabolites, such as proline (Pro), Glu, serine (Ser), and alanine (Ala), in wild soybean seedlings compared to semi-wild and cultivated soybean has been observed [85]. In response to 250 mM NaCl salt stress, the level of Asp increased by 11-fold in the root and about 6.2-fold in the shoot of *Aeluropus lagopoides* [86]. Under the same conditions, Asn, Lys, glycine (Gly), and Pro increased by 1.46- to 9.98-fold in the shoot, while in the root, Gly, Pro, Phe, and ethanolamine increased by approximately 2.5- to 15.6-fold. NaCl-treated wheat seedlings showed a 15.75-fold increase in Asp, and a 1.6-fold increase in total free amino acids compared to the control. Likewise, there was a significant enhancement (2.7-fold) of Asp after plants were inoculated with *Bacillus amyloliquefaciens* RWL-1 under salinity stress conditions [87]. The high accumulation of Asp and other amino acids, such as Pro under salt stress, has played an essential role in plants in highly saline conditions by maintaining the intracellular osmotic potential and stabilizing membrane proteins [88]. Furthermore, the change of Asp content has been reported to be coupled with the alteration of protein metabolism in salt-stressed plants [89].

In the same manner, the response of Asp to cold stress has been observed. For instance, the level of Asp together with Pro and putrescine increased rapidly in leaves of strawberry during cold (2 °C) acclimation processes [90]. In fig fruits during cold storage, the contents of Asp, as well as Glu, were upregulated, while the level of most other free amino acids decreased [91]. A heatmap matrix of a Pearson’s correlation coefficient test reveals that the enhancement of Asp and Glu is positively correlated to water loss, glucose, and fructose variables [91]. Likewise, the Asp content in rye substantially responded to cold hardening. A larger amount of Asp was found in the variety with higher frost tolerant ability, especially in the early phases of cold acclimation. The contents of Asp, Pro, Tyr, and glycine betaine were observed to linearly increase in response to overwintering (cold stress) [92]. In addition, a greater accumulation of Asp, Glu, and β-alanine in leaves, concomitant with an enhancement of raffinose and 1-kestose in roots of wheat, has been demonstrated to be associated with the improvement of phosphorus use efficiency (PUE) in P-efficient wheat cultivars under low P supply [93]. These results clearly show the active response and co-regulation activity of Asp and coupled amino acids, sugars, and organic acids to stresses, suggesting that modulation of metabolite flux from Asp is likely beneficial for plant stress adaption, as it provides the plant with essential metabolism substances and energy.

Oxidative stress is a common consequence for plants exposed to non-optimal environmental conditions. To cope with oxidative stress, plants employ the redox buffer system, scavenging enzymes, and metabolic mechanisms to detoxify reactive oxygen species (ROS) [94,95]. It has been found that the amounts of Asp, as with malate, 2-OG, Glu, and hexose phosphates, were decreased in *Arabidopsis* roots treated with menadione to elicit oxidative stress. On the contrary, these compounds increased and returned back to the control levels following the removal of menadione [96]. The reactive and recovery responses of Asp and derived compounds to oxidative stress are thought to be pivotal for reconfiguring the metabolic network to help plants recover and survive [97].

### 3.5. Asp Acts as a Biomarker of Biotic Stress and Environment-Induced Exposure

As biochemically active compounds, free amino acids strongly respond to increased amounts of toxic substances in the environment [84]. In agreement with this, the free amino acid content increased in tomato plants grown in soil contaminated with arsenate (As(V)). Among these free amino acids, Asp and Glu were intensively accumulated [98]. The content of Asp was significantly reduced in aluminum (Al)-treated citrus roots, although most of the amino acids, as well as some sugars (i.e., raffinose and trehalose), were increased [99]. Asp, together with other amino acids relating to nitrogen metabolism, showed high accumulation in response to the arbuscular colonization of *Medicago truncatula* Gaertn. cv. Jemalong (A17) [100]. The level of Asp concentration was reported to be elevated in the root, stem, and leaf tissue of Fusarium wilt-symptomatic watermelon compared to the asymptomatic plants. As a consequence, it promotes the growth and development of *Fusarium oxysporum* f. sp. niveum. [101]. In addition, a higher concentration of Asp was detected in tomato seedlings grown from seeds primed with 100 nM jasmonic acid (JA) treatments under nematode infection, displaying the vital role of Asp in the fight against nematodes during JA treatment in tomatoes [102]. However, disease suppression of Fusarium crown rot showed a positive change of Asp in mycorrhizal asparagus, indicating the possible implication of Asp in *Fusarium* infection [103].

Taken together, notable stress-related responses of Asp to variable environmental exposures in plants have been observed, indicating an essential role of Asp in response to stress (Table 1). Since Asp is a key precursor for the biosynthesis of many fundamental metabolites, it can be reasonably inferred that the change of concentration of Asp fine-tunes the availability of downstream metabolites that are indispensable for plants to grow and to counteract various stresses. For instance, it fine-tunes central metabolism with glycolysis (sucrose, hexose, pyruvate, etc.), the citric acid cycle (2-OG, succinate, etc.), NAD, and nucleotides to support cell survival [9,11,78,79,83,90,91,96]; the essential amino acids Met, Lys, Thr, Ile, and other amino acids to adjust the total amino acid pool and protein metabolism [89,104]; and the organic N carriers Glu, Asn, and Gln to regulate N mobilization, storage, and recycling [71,72,105,106,107]. In addition, alteration of Asp levels can also be linked with malate activity as a consequence of stomatal opening, Ca^2+^ uptake inhibition, and amino acid transformation [74,86]. In addition, increasing Asp concentration in plants during anaerobic stress decreases the cytoplasmic pH, which affects the production of other intracellular metabolites, such as alanine and GABA [108].

## 4. Asp Signaling and Its Association with Phytohormones

Signaling in plants has recently received much attention in most research areas, but this is yet to be realized for Asp. Thus far, little is known about the plant Asp receptor. In *E. coli*, the Asp receptor, a class of cell-surface signal-transducing proteins, has been characterized. It transduces the transmembrane signal to the cytoplasm, mediating chemotaxis in bacteria, or adjusting metabolic functions, growth, differentiation, and division in eukaryotic cells [110,111,112,113,114]. Recently, one of the methyl-accepting chemotactic proteins, *Tlp1* (Tlp stands for transducer-like protein), in *Campylobacter jejuni* has been found to encode the Asp receptor (named CcaA). It only accepts Asp but no other amino acid as its ligand [113]. External binding of Asp triggers changes affecting both the internal signaling site and the internal adaptation site of the Asp receptor [115].

Though Asp receptors have not been characterized in plants, the Asp and phytohormone interconnection, in general, has been proposed to mediate different developmental processes in a plant’s life cycle through their signal pathways. Ethylene is a phytohormone exerting multiple functions modulating plant growth and senescence. Ethylene biosynthesis depends directly on the Asp-derived amino acid pathway in that the ethylene biosynthesis precursor, 1-aminocyclopropane-1-carboxylate (ACC), is produced through and regulated by Asp metabolism [116]. Moreover, ACC synthase (ACS), the key rate-limiting enzyme of ethylene biosynthesis [117], similar to AspAT, is a pyridoxal-phosphate (PLP)-dependent enzyme clustered into subgroup I of the α family of aminotransferases [118,119]. Further close examination of the ACS protein by X-ray structure analysis discloses its functional similarity in terms of amino acids embracing the catalytic site to the AspAT counterpart [120,121]. Accordingly, ACS10 and ACS12 show broad specificity for Asp and aromatic amino acids [122]. Thus, it is not difficult to infer the central role of Asp and AspAT in ethylene biosynthesis and signaling pathways [123]. This point of view has been confirmed by the observation that disturbance of ethylene biosynthesis by exogenous application of the ethylene biosynthesis inhibitor α-aminoisobutyric acid (AIB) shows a linear correlation between the level of Asp and the change in the root length and shoot surface area [116]. Hence, Asp could be a key modulator of the ethylene and/or ethylene signaling pathway, which probably aids plant root and shoot development in an ethylene-dependent way.

Indole-3-acetic acid (IAA)–amino acid conjugate synthesis pathways are among the important regulatory mechanisms that control auxin activities during physiological and pathophysiological responses. Several group II genes of GRETCHEN HAGEN3 (GH3) encode IAA–amido synthetases in plants influence IAA homeostasis via IAA conjugation pathways [124]. The IAA-conjugating pathways are entangled with most amino acids, among which conjugation of IAA to Asp moieties, producing IAAsp, is predominant in many plants [124,125,126]. In vitro assays in *Arabidopsis* showed that IAAsp is the primary conjugate of IAA formed by GH3 genes, and exogenous application of IAA induces the conversion of IAA to IAAsp [127]. A feeding experiment using labeled IAA shows that *de novo* synthesis of IAAsp is the main route operating in conifer seedlings [124]. In *Vitis vinifera* L., towards the onset of the ripening of grape berries, an increase in IAAsp accumulation, along with low concentrations of IAA, has been observed [128]. It is worth noting that the conjugation pathway of IAAsp is fast and inducible. This allows plants to effectively control auxin homeostasis upon developmental and stress cues [129,130]. Although, generally, IAAsp is known as an intermediate for auxin degradation, it has been established that *Medicago truncatula* hydrolase can hydrolyze IAAsp to generate free IAA [131,132]. Consistently, IAAsp treatment increases the root length of pea [133]. IAAsp is also involved in the carbonylation of protein and can therefore regulate tissue response to abiotic and biotic stresses [129,130,134]. In addition, the concentration of IAAsp increased more than twofold upon NaCl stress (200 mM) in Chinese cabbage seedlings [135].

Although some basics on Asp signaling and interconnection with phytohormones (i.e., ethylene and auxin) have been described, the recognition and/or signal transduction mechanisms of Asp remain mostly unknown. Further metabolomics analysis and mutant and transgenic approaches will be helpful to identify genes associated with Asp signaling and elucidate its mechanism in plants.

## 5. Conclusions and Future Perspective

The amino acid Asp serves as an important metabolic hub for the biosynthesis of many metabolites, including the Asp family amino acids (essential amino acids), Arg, Glu, Asn, aromatic amino acids (Tyr and Phe), nucleotides, proteins, TCA cycle intermediates, glycolysis pathway intermediates, NAD, and hormonal conjugates, that are imperative for plant growth and development, and for plants to react to abiotic stress and defense. Regulation of Asp content, fluxes, and transport through the plant is thus critical for plant adaptation to fluctuating environmental conditions. Accordingly, allocation and interconversion of Asp within plants are a feasible strategy to increase plant acclimation abilities. However, to date, the biological principle controlling the biosynthesis and catabolism of Asp under physiological and stress states has been poorly addressed. Further investigation is required to elucidate the underlying metabolic and molecular mechanisms in the biological processes of Asp uptake, allocation, assimilation, and homeostasis, to gain deep insights into the mode of action of Asp.

The recent increasing effort in plant genome sequencing and the extensive use of genetic engineering tools will enable researchers to study the various pathways of Asp metabolic flux extensively, and the enzymes involved in specific plants. This will advance the study of Asp metabolism, transport, and stress signal integration and, consequently, aid researchers to model the Asp metabolic network and target specific enzymes in a specific tissue or subcellular compartment to precisely modulate the metabolic flux of Asp.

## Figures and Tables

**Table 1:** Induction and repression of Asp in different plant species under various stress conditions.

Stress	Species	Tissues (Stress Period)	Asp Fold Change	Change of Asp-Associated Metabolites	Physiological Role	Ref.
Drought	*Astragalus membranaceus*	Roots (10 days)	2.3	↑Asp family metabolism, ↑glutamate, ↑GABA,↑TCA cycle, ↑sucrose	Sensing water status	[78]
	*Cicer arietinum* L. (chickpea)	Leaves	−2.5~−6.1	↑Thr, ↑Met, ↓Asn, ↑citrulline	Osmoregulation	[81]
	*Caragana korshinskii*	Leaves and roots	−0.32~−0.63	↑Asn, ↑sugars/glycosides, ↓Glu,↓isocitric acid	Drought-responsive metabolites	[83]
	Triticeae	Roots and leaves	>2	↑Succinate, ↑Trehalose, ↑Glu, ↑Asn, ↑Met, ↑Phe	Drought stress-specific responsive metabolites	[79]
	*Brassica oleracea* L. var. *acephala* (kale)	Leaves	−1.3	↓Glu, ↓Thr, ↓Ala, ↑Pro	Biomarker for drought tolerance	[82]
Salinity	*Aeluropus lagopoides*	Shoots and roots	6.2~11	↑Asn, ↑Lys, ↓malate	Stomatal opening, inhibited Ca2+ uptake	[86]
	Wheat	Seedlings (17 days)	15.75	↑Ile, ↑Lys, ↑Phe, ↑Pro, ↓Glu, ↓Arg, ↓Met	Protein metabolism, osmoprotection	[89]
N starvation or low N	Non- nodulated soybean	Phloem sap (4 days)	−3.7	↓Asn, ↓Glu, ↑malate, ↑GABA	Transform to malate to deliver the amino acids	[74]
	Maize	Leaves	≈2	↓Asn, ↓Glu	Regulation of N mobilization	[72]
	*Solanum tuberosum* L. (potato)	Shoots and tubers of potato cv. Kufri Jyoti	>5	↑Thr, ↑Asn, ↑Glu,	NUE efficiency	[75]
	Tobacco	Leaves	>−2	↑Glu, ↑Lys, ↑Ile, ↓Gln, ↓Arg, ↓Phe	Represents a significant proportion of the total amino acid pool	[104]
	Soybean	Xylem sap	≈8	↓Asn, ↓Gln, ↑Glu, ↑Ala, ↑GABA	N recycling, source of N in alanine formation	[71]
Supplementation of nitrate	Soybean	Roots	≈3	↑Asn, ↑Glu, ↑Gln	Provide C skeleton for the synthesis of Asn	[105]
Low C	Tobacco	Leaves	>−2	↑Glu, ↑Asn, ↓Phe	Represents a significant proportion of the total amino acid pool	[104]
Light	Sunflower	Leaf discus	≈2	↑Glu, ↑Gln	Convert to Asn for N storage and transport in the dark	[106]
	Tobacco	Leaves	2.6	↑Phe	Light-responsive marker metabolites	[104]
Cold	*Fragaria* × *ananassa* (strawberry)	Leaves and roots of Duch. “Korona”	3–5	↑Ile, ↑hexoses, ↑pentoses	Protective metabolites	[90]
	*Secale cereale* (rye)	Plant crown	3	↑Glu, ↑Pro	Frost tolerance improvement	[92]
	*Ficus carica* L. (fig)	Fruits	>2	↑Glu, ↑Glucose, ↑fructose, ↓Arg, ↓GABA, ↓Phe, ↓Ile, ↓Pro	Cold-responsive marker metabolites	[91]
Low P	*Triticum aestivum* L. (Wheat)	Leaves	1.2	↑Gln, ↑β-alanine, ↑raffinose, ↑1-kestose	Enhanced PUE	[93]
Fusarium wilt	*Citrullus vulgaris* (watermelon)	Leaves, stems, and roots	33–43	↑Lys, ↑Arg, ↑citrulline	Biomarker of Fusarium wilt disease	[101]
Fusarium crown rot	*Asparagus officinalis* L., cv. “Welcome”	Mycorrhizal asparagus shoots	≈1.7	↑Glu, ↑Arg, ↑citrulline, ↑GABA	Disease tolerance	[103]
Parasitic weed	Faba bean	Tubercles of tolerant line	≈−0.4	↓Asn, ↓Glu, ↓Gln, ↓GABA,↓sucrose	N metabolism of the parasite	[107]
Arbuscule	*Medicago truncatula*	Mycorrhizal roots	>10	↑Glu, ↑Asn, ↑Gln, ↑sucrose, ↑trehalose	Associated with higher N availability	[100]
JA (100 nM)	Tomato	Seedlings	1.6	↑Asn, ↑Glu, ↓Gln,↓Lys, ↓Met,↓Arg	Osmoregulation	[102]
Oxidative stress	*Arabidopsis thaliana*	Roots (6 h)	≈2	↓Glu, ↓malate, ↓succinate, ↓fumarate, ↓hexose phosphates, ↑2-OG, ↑pyruvate, ↑citrate	Oxidative stress-responsive metabolites	[96]
Hypoxia	Muskmelon	Roots (6 days)	1.23	↑Thr, ↑Glu, ↑Lys, ↑GABA	Hypoxia-responsive metabolites	[109]
Anoxia	Rice	Excised roots	≈−2	↑GABA, ↑Pro, ↑pyruvate,↓Glu,↓Gln, ↓Asn, ↓2-OG	Corresponds to a weak fall in cytoplasmic pH	[108]
Arsenate (As(V))	Tomato	Aboveground tissues and roots	2.4–3.1	↑Asn, ↑Gln, ↑Glu, ↑Arg, ↑Lys, ↑Ile	Marker for As(V) stress	[98]
Aluminum (Al)	Trifoliate orange	Roots	−2	↓Ile, ↓Glu, ↓malate,↓sugars, ↑Asn, ↑Lys, ↑Gln	Marker for Al stress	[99]

↑, upregulation; ↓, downregulation; Asp, aspartate; Glu, glutamate; Gln, glutamine; Arg, arginine; Ile, isoleucine; Pro, proline; 2-OG, 2-oxoglutarate; GABA, γ-aminobutyric acid; JA, jasmonic acid.

## Data Availability

Not applicable.
